# Combination of UGT1A1 polymorphism and baseline plasma bilirubin levels in predicting the risk of antipsychotic-induced dyslipidemia in schizophrenia patients

**DOI:** 10.1038/s41537-024-00473-1

**Published:** 2024-05-17

**Authors:** Chenquan Lin, Shuangyang Zhang, Ping Yang, Bikui Zhang, Wenbin Guo, Renrong Wu, Yong Liu, Jianjian Wang, Haishan Wu, Hualin Cai

**Affiliations:** 1 Department of Pharmacy, The Second Xiangya Hospital of Central South University, Changsha, China; 2 Institute of Clinical Pharmacy, Central South University, Changsha, China; 3 Department of Psychiatry, Hunan Brain Hospital, Changsha, China; 4 International Research Center for Precision Medicine, Transformative Technology and Software Services, Hunan, China; 5 Department of Psychiatry, The Second Xiangya Hospital of Central South University, Changsha, China; 6 National Clinical Research Center on Mental Disorders, Changsha, China

**Keywords:** Biomarkers, Schizophrenia

## Abstract

The prolonged usage of atypical antipsychotic drugs (AAPD) among individuals with schizophrenia often leads to metabolic side effects such as dyslipidemia. These effects not only limit one’s selection of AAPD but also significantly reduce compliance and quality of life of patients. Recent studies suggest that bilirubin plays a crucial role in maintaining lipid homeostasis and may be a potential pre-treatment biomarker for individuals with dyslipidemia. The present study included 644 schizophrenia patients from two centers. Demographic and clinical characteristics were collected at baseline and 4 weeks after admission to investigate the correlation between metabolites, episodes, usage of AAPDs, and occurrence of dyslipidemia. Besides, we explored the combined predictive value of genotypes and baseline bilirubin for dyslipidemia by employing multiple PCR targeted capture techniques to sequence two pathways: bilirubin metabolism-related genes and lipid metabolism-related genes. Our results indicated that there existed a negative correlation between the changes in bilirubin levels and triglyceride (TG) levels in patients with schizophrenia. Among three types of bilirubin, direct bilirubin in the baseline (DBIL-bl) proved to be the most effective in predicting dyslipidemia in the ROC analysis (AUC = 0.627, *p* < 0.001). Furthermore, the odds ratio from multinomial logistic regression analysis showed that UGT1A1*6 was a protective factor for dyslipidemia (ß = −12.868, *p* < 0.001). The combination of baseline DBIL and UGT1A1*6 significantly improved the performance in predicting dyslipidemia (AUC = 0.939, *p* < 0.001). Schizophrenia patients with UGT1A1*6 mutation and a certain level of baseline bilirubin may be more resistant to dyslipidemia and have more selections for AAPD than other patients.

## Introduction

Schizophrenia as a complicated and severe mental disease, influences about 1% of the population around the world^1^. Atypical antipsychotic drugs (AAPD) are the mainstay of the treatment and can induce metabolic side effects like weight gain, dyslipidemia, hyperglycemia, insulin resistance, etc. And these are closely related to the development of metabolic syndrome (MetS) in patients with schizophrenia^2^. However, in contrast to extra-pyramidal symptoms side effects^3^, there was little evidence to suggest a dose-response relationship between lipid metabolism disorder and most antipsychotic medications^4,5^. Therefore, how to predict the risk of somatic comorbidity before treatment and improve the precision medication with AAPDs become one of the issues to be urgently resolved in clinical practice.

The progesterone receptor membrane component 1 (PGRMC1) is a multi-protein complex that can directly bind to heme and associate with cytochrome P450 proteins, the EGFR receptor tyrosine kinase, the RNA-binding protein PAIR-BP1, and other effect proteins so that PGRMC1 is involved in various functions like tumor growth, cholesterol synthesis, and drug metabolism^6–9^. Besides, it has been reported that PGRMC1 can interact with the lipid regulatory proteins like insulin-induced gene (INSIG) and SREBP cleavage activating protein (SCAP), which play the role in triglyceride and cholesterol synthesis^10^. When INSIG is inhibited, SCAP is released to escort SREBP, sterol regulatory element-binding protein, to the Golgi for processing the lipogenesis and cholesterogenesis^11^. Based on the important role of PGRMC1/INSIG/SCAP/SREBP pathway in lipid regulation, a previous study of our team hypothesized that AAPD may interact with the upstream of PGRMC1 and influence the following changes in lipid metabolism^12^. We found that with clozapine or risperidone treatment, there was an increase in both triacylglycerols and total cholesterol levels, achieved through the inhibition of PGRMC1/INSIG-2 and activation of SCAP/SREBP expressions. Therefore, inhibition of PGRMC1/INSIG/SCAP/SREBP represents the potential mechanism of lipid disturbance induced by AAPD. However, studies on the polymorphism of PGRMC1 are scarce, mounting evidence focuses more on the polymorphism of INSIG and SREBP. Among the 160 schizophrenia patients in German, Hellard^13^ et al found that there was a strong association between three single nucleotide polymorphisms in INSIG2 (rs17587100, rs10490624, and rs17047764) and antipsychotic-related weight gain. Li et al.^14^ detected the SREBP2 gene in 621 schizophrenia patients under clozapine treatment and found that rs1052717 and rs2267443 were related to MetS, especially individuals carrying A bases that were prone to MetS.

For a long time, different forms of bilirubin including total bilirubin (TBIL), direct bilirubin (DBIL), and indirect bilirubin (IBIL) are considered waste products of heme metabolism. Recent updates demonstrated that bilirubin can be an important endogenous antioxidant against tissue oxidative damage and a physiological regulator of chronic inflammation^15^, which are closely related to lipid metabolism disorder^16^. Interestingly, the bilirubin itself is not merely a metabolite but exerts pivotal effects on lipid metabolism through peroxisome proliferator-activated receptor-alpha (PPARα) signaling^17^. The bilirubin can directly bind and activate the downstream-regulated genes of PPARα to improve its transcriptional activity, resulting in increased expression of fat-regulating genes carnitine palmitoyltransferase I (CPT1)^18^ and fibroblast growth factor 21 (FGF21)^19^ and subsequently decrease lipid synthesis and promote energy metabolism^20^. Besides, there is an indirect influence of bilirubin via heme on lipid metabolism. Firstly, PGRMC1 can interact with ferrochelatase (FECH) which is responsible for the metabolism of heme, the precursor of bilirubin^21^. Meanwhile, free heme can bind to PGRMC1 and sequentially be transmitted to the endoplasmic reticulum, then to the progesterone receptor membrane component 2 (PGRMC2) on the nuclear membrane, and finally enter into the nucleus to regulate REV-ERB alpha (Rev-Erbα) and BTB and CNC homology 1 (BACH1), which will drive the changes in mitochondrial energy metabolism in brown adipose tissue^22^. Therefore, the level of bilirubin can be an important factor in the regulation of lipid metabolic status. In support, a meta-analysis has demonstrated that there is an inverse association between serum bilirubin levels and the risk of adverse metabolic outcomes, and this association is slightly stronger in men than in women^23^. However, IBIL and DBIL, the components of TBIL, were not analyzed separately in this study so it was tough to evaluate which bilirubin fraction was associated with which metabolic outcomes.

**Fig. 1:**
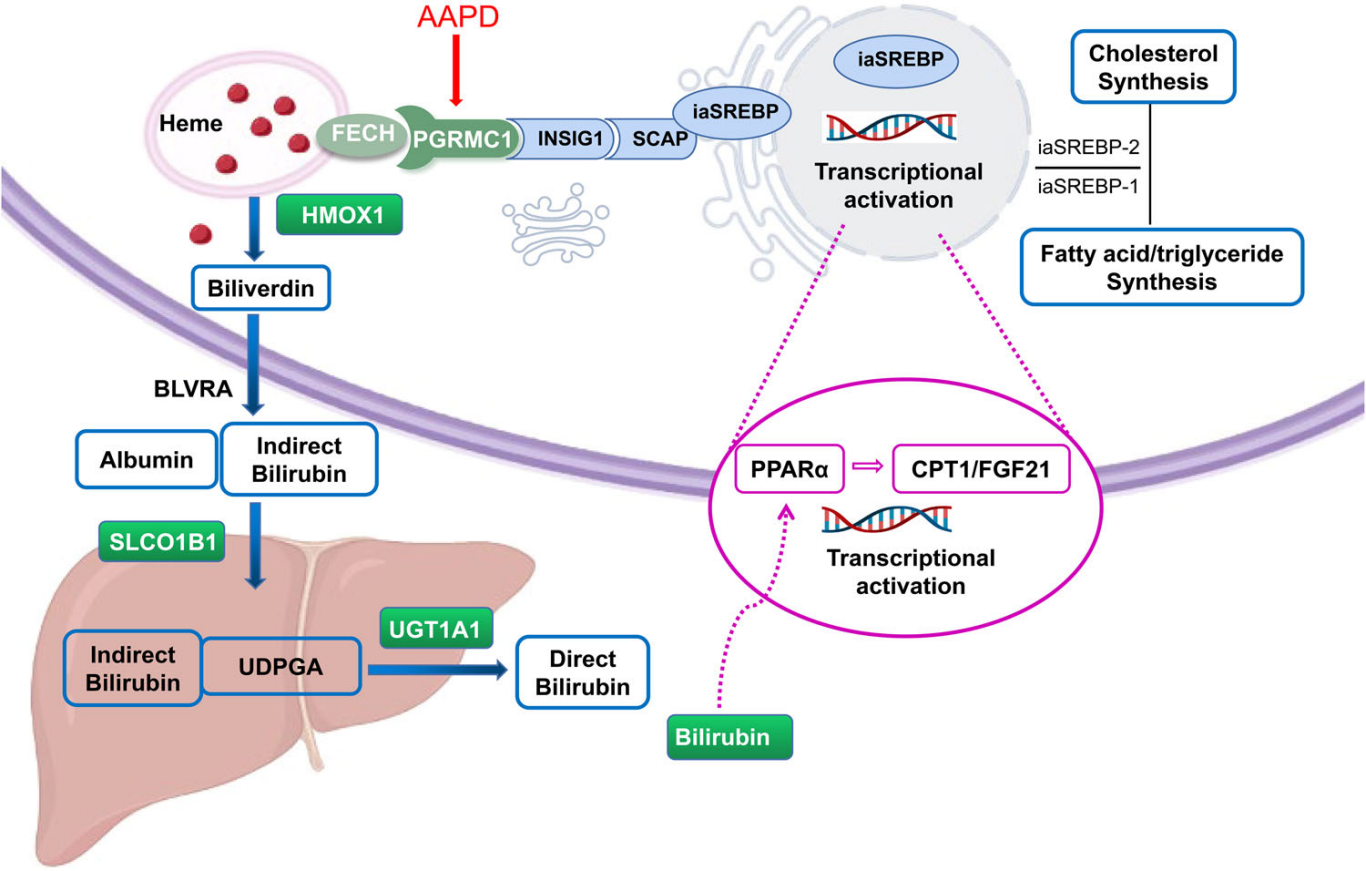
Pathway of bilirubin metabolism and lipid metabolism. FECH ferrochelatase, PGRMC1 progesterone receptor membrane component 1, INSIG1 insulin-induced gene 1, SREBP sterol regulatory element-binding proteins, SCAP SREBP cleavage activating protein, HMOX1 heme oxygenase 1, BLVRA biliverdin reductase A, UDPGA uridine diphosphate glucuronic acid, PPARα peroxisome proliferator-activated receptor-alpha, CPT1 carnitine palmitoyltransferase I, FGF21 fibroblast growth factor 21, UGT1A1 UDP-glucuronosyltransferase 1A1.

Based on the function of bilirubin in lipid metabolism, we attempt to explore the targets in the bilirubin metabolic pathway, which may correlate and influence the level of lipid parameters. As shown in Fig. 1, heme oxygenase 1 (HMOX1)^24^ is one of the rate-limiting enzymes in bilirubin metabolism, and is responsible for the catabolism of heme. Studies have shown that the (GT)n polymorphism, repeated sequence of the Guanosine thymidine dinucleotide (GT) on the microsatellite promoter from HMOX1 (HO-1) enzyme affects the extent of oxidative stress in chronic hemodialysis patients and is associated with the occurrence and death of cardiovascular disease^25,26^. The solute carrier organic anion transporter family member 1B1 (SLCO1B1)^27^ is a liver-specific transporter responsible for the transport of IBIL to the liver. Studies have reported that SLCO1B1 gene mutation is one of the independent predictors of serum bilirubin level. For example, the elevation of bilirubin induced by the SLCO1B1*15 gene mutation is intimately associated with hyperbilirubinemia (Gilbert syndrome, GS)^28^. UDP-glucuronosyltransferase 1A1 (UGT1A1)^29^ is another important rate-limiting enzyme in bilirubin metabolism, mainly responsible for converting fat-soluble bilirubin into a water-soluble form for further excretion. It is reported that polymorphisms in the promoter (TA)n of the UGT1A1 gene can result in Gilbert syndrome (a benign unconjugated bilirubinemia)^30^. Furthermore, A review recently supported that the abnormal activity of UGT1A1 not only has a tight correlation with Gilbert syndrome and Crigler–Najjar syndrome but also may be related to bilirubin-induced encephalopathy, Alzheimer’s disease, hepatobiliary diseases, cholestasis and some other diseases, among most of which are due to bilirubin metabolism^31^. Whereas, patients with low activity of UGT1A1 are hard to be detected in daily life.

To date, the interrelationship between bilirubin metabolism and lipid metabolism in schizophrenia patients remains to be elucidated. Levels of bilirubin in humans are highly heritable so genes involved in bilirubin metabolism are important candidate genes to control the serum concentration of bilirubin^32^. We hypothesize that through the inhibition of PGRMC1 by AAPDs, (1) the metabolic regulation of bilirubin and lipid may be dynamically linked, and (2) in patients with schizophrenia, the risk of developing post-treatment related lipid metabolic disorder may be predicted by the combination of the variations in the baseline bilirubin levels and bilirubin-related genotypes.

## Material and methods

### Ethics statement

This study (Chinese Clinical Trial Registry: ChiCTR2100048968) was approved by the Medical Ethics Committee of the Second Xiangya Hospital of the Central South University and Hunan Province Brain Hospital, and all protocols were performed under the guidelines of the Helsinki Declaration^33^. The patients whose blood samples were collected for genotyping provided written informed consents before participating in this study.

### Participants

We retrospectively collected the clinical data of patients before and after AAPD treatment from the Second Xiangya Hospital of Central South University (center 1) and Hunan Province Brain Hospital (center 2) from July 2021 to February 2022 to explore the intrinsic interaction among potential indexes. Meanwhile, the patient blood samples from Hunan Province Brain Hospital (center 2) were selected to conduct genotyping. Inclusion criteria were as follows: (1) patients diagnosed with schizophrenia according to the Diagnostic and Statistical Manual of Mental Disorders, Fifth Edition (DSM-5) diagnostic criteria, (2) the age ranged from 18 to 65, (3) no hepatitis B or C, (4) under AAPD treatment for over two weeks after admission, and (5) off AAPD medication for at least one month before admission. Exclusion criteria included: (1) pregnant woman, (2) patients with severe liver diseases such as liver cirrhosis, drug-induced liver injury, and liver cancer, etc, (3) patients without laboratory test results, (4) patients with a history of smoking or/and alcohol abuse, or (5) patients with abnormal liver biological function like glutamic-pyruvic transaminase (ALT) or glutamic-oxalacetic transaminase (AST) > 100IU/L, TBIL > 34.2 μmoL/L (clinical manifestations is jaundice)^34,35^. When patients were admitted to hospital and enrolled in our study, they would be cared for in non-smoking ward and kept light diet. Since biomarker changes can vary with the number of episodes and duration of disease^36,37^, the patients in this study were divided into three subgroups: first episode, 2–3 episodes, and over 4 episodes. According to patient messages from the Electronic Medical Record, the relapse is defined as any of the following: readmission due to a relapse episode; a 25% increase in PANSS total score and an increase in the level of psychiatric care during hospitalization; intentional self-harm; suicidal or homicidal ideation deemed clinically significant by the researcher’s judgment; violent behavior causing clinically significant harm to others or property^38,39^.

In our study, lipid metabolism disorder could be confirmed when patients meet three or more criteria of the following, including at least two of the major criteria as follows^40^: (1) Triglycerides (TG) was higher than 1.7 mmol/L, (2) High-density lipoprotein cholesterol (HDL-CH) was lower than 1.3 mmol/L, (3) Cholesterol (CHOL) was above 5.18 mmol/L, (4) Low-density lipoprotein cholesterol (LDL-CH) was higher than 3.12 mmol/L, and one of the auxiliary criteria such as: (1) body mass index (BMI) was over 25 kg/m^2^, and (2) systolic blood pressure (SBP) was above 130 mmHg and/or diastolic blood pressure (DBP) was above 85 mmHg.

### Retrospective cohort

Patient cases from center 1 were used mainly as a retrospective cohort study without sample genotyping. Baseline and 4-week post-treatment data were collected, including (1) demographic parameters such as sex, age, disease duration, admission frequency, height, and weight; (2) medication details, including drug types, doses, administration times, and monotherapy or polypharmacy; (3) blood routine parameters such as white blood cell count, red blood cell count, hemoglobin, and platelet counts; (4) liver function indexes such as glutamic-pyruvic transaminase (ALT), glutamic-oxaloacetic transaminase (AST), total bilirubin (TBIL), direct bilirubin (DBIL), indirect bilirubin (IBIL), and total protein (TP); (5) lipid metabolism parameters including triglycerides (TG), high-density lipoprotein cholesterol (HDL-C), total cholesterol (TC), and low-density lipoprotein cholesterol (LDL-C); and (6) other important parameters, such as medical history, smoking and/or drinking habits, and family history. Our objective was to investigate the distribution characteristics of demographic parameters, biochemical parameters, and lipid-related parameters in three subgroups based on different episodes and explore the potential relationship between bilirubin and lipid parameters.

### Prospective cohort

In this cohort, we expanded the study to include patient cases from Center 2 to confirm the findings from Center 1. We also collected blood samples from the same patient cases for further genotyping. Among the alternative genotypes, we selected two SNPs from UGT1A1 (rs34983651 and rs4148323) and two SNPs from SLCO1B1 (rs2306283 and rs4149056) in bilirubin-related metabolism, as well as INSIG1 rs9769826, INSIG2 rs7566605, SREBP1 rs2297508, SREBP2 rs1052717 in lipid-related metabolism as the main loci. However, the mutation frequencies of PGRMC1 and HMOX1 in Asian people are very low so exon testing was considered. Based on the results of genotyping, we aimed to explore the effect of different genotypes on bilirubin indexes and biomedical indexes and to identify the potential genetic biomarkers for predicting dyslipidemia.

### Genotyping

In the present study, multiplex PCR target capture technology (mPCR) was used to construct the library and sequence. Firstly, we extracted the DNA from the marked blood samples. Then, we used Qubit® 3.0 Fluorometer (Thermo, Q33216) to detect the concentration of DNA samples. In general, the OD value is ranged from 1.8 to 2.0 while the total DNA is over 40 ng. The DNA samples that accorded with the criterion of the genomic library were used for the subsequent sequencing. The specific process of library inspection included 1 μL of the library for quantification of about 10–50 ng/μL using Qubit dsDNA HS Assay Kit and 1 μL of the sample using the Agilent 2100 Bioanalyzer system (Agilent DNA 1000 Kit) to determine the length of the library. The length of the library is about 300–450 bp. Finally, a high-throughput sequence platform (Illumina HiSeq2500/NextSeq500) was used for sequencing. The acquired original sequenced reads must be via the standard quality assessment including the comparison rate, coverage rate, capture rate, uniformity, and the distribution of SNPs from sequenced reads should accord with the Hardy–Weinberg equilibrium (*p* > 0.05). After the quality assessment, the SNPs on patients from clear reads could be used in the subsequent study.

### Statistical analyses

All statistical analyses were operated by SPSS (version 26, IBM), and figures of analysis were presented by GraphPad Prism (version 8.0.1). In our retrospective cohort, the Kolmogorov–Smirnov test (K–S test) was used to determine the normal distribution of the data. The Bartlett test or Levene test was used to test the homogeneity of variances. Parametric data were compared by one-way analysis of variance, while the non-parametric data were compared by the Kruskal–Wallis *H* test. We performed the methods above to explore differences in biochemical parameters from subgroups of different episodes, and the effect of AAPD on bilirubin levels in schizophrenia patients. The Pearson correlation analysis was performed to explore the relationship between bilirubin and lipid metabolism parameters. To deal with the missing data, variables with a missing rate exceeding 15% were considered for exclusion before the statistical analyses. For variables with a lower missing rate, the random forest method was applied for imputation when necessary.

In our prospective cohort, the one-way analysis of variance was used to compare levels of bilirubin or lipid metabolism parameter across each genotype or the Kruskal–Wallis *H* test instead when the samples wouldn’t follow the rules of one-way analysis of variance. Furthermore, the effects of bilirubin and bilirubin-related gene mutations on lipid disturbances were observed by multinomial logistic regression. The odds ratio indicated protective or risk factors for dyslipidemia after medications. Finally, the receiver operator characteristic curve (ROC) was used to cooperate with the potential factors to measure the diagnostic performance of dyslipidemia. Values were expressed as mean ± standard deviation or *N* (%). A two-tailed level of 0.05 was accepted to be statistically significant.

## Results

### Demographics

**Fig. 2:**
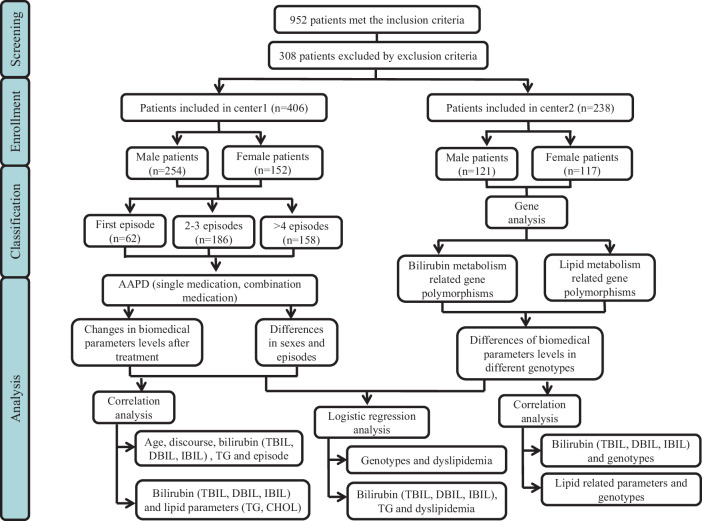
Participant flow according to inclusion criteria and exclusion criteria. TBIL total bilirubin, DBIL direct bilirubin, IBIL indirect bilirubin, TG triglyceride.

The flow chart about how the study was conducted and related methodology is depicted in Fig. 2. Our study recruited 601 patients with schizophrenia from the Second Xiangya Hospital (center 1) and 351 from the Hunan Brain hospital (center 2). After screening according to the criteria listed above, 644 patients (Center 1: 406, Male/Female 254/152; Center 2: 238, Male/Female 121/117) were finally included for further clinimetric and genotype data analyses. The demographic characteristics of patients with schizophrenia from two centers were summarized in Table 1. No significant changes were observed in sex (*p* = 0.92), BMI (*p* = 0.208), CHOL (*p* = 0.75), LDL-CH (*p* = 0.372), or chlorpromazine-equivalent dose (*p* = 0.479) within the first episode, 2–3 episodes and over 4 episodes, whereas the TBIL (*p* = 0.009), DBIL (*p* = 0.015), IBIL (*p* = 0.003) and TG (*p* < 0.001) were shown significant differences among three subgroups. In our retrospective cohort (center 1), the olanzapine (46.8% in the first episode, 44.6% in 2–3 episodes, 22.9% in over 4 episodes) was used more frequently and the combination treatment followed behind. The combination treatment was mostly used in relapsed subgroups (54 in 2–3 episodes, 61 in over 4 episodes). Moreover, as presented in Table 1, the result of the prospective cohort (center 2) also confirmed the clinical characteristics of center 1.

**Table 1:** Demographic and clinical characteristics of schizophrenia patients from two centers.

characteristics	Center1			Center2			F/χ^2^	*P
	First episode	2–3 episodes	>4 episodes	First episode	2–3 episodes	>4 episodes		
*N*	62	186	158	57	104	77		
Male (%)	40 (64.5%)	105 (56.5%)	109 (69.0%)	25 (43.9%)	55 (52.9%)	41 (53.2%)	4.781	0.92^a^
Disease course (years)	1.11 ± 1.7	5.77 ± 6.1	9.91 ± 6.8	1.92 ± 4.8	5.87 ± 4.5	10.9 ± 6.7	243.9	<0.001^b^
Age (years)	27.4 ± 10	29.2 ± 9.7	30.8 ± 10	28.6 ± 11	31.3 ± 11.4	33.9 ± 9.3	5.64	0.004^c^
BMI (kg/m^2^)	22.1 ± 8.7	23.1 ± 8.5	22.2 ± 3.8	20.7 ± 5.1	21.7 ± 3.1	23.5 ± 4.9	1.58	0.208^c^
TBIL (μmol/L)	13.9 ± 6.2	13.4 ± 6.7	11.5 ± 5.3	13.9 ± 6.9	14.8 ± 7.7	13.7 ± 7.3	4.78	0.009^c^
DBIL (μmol/L)	4.77 ± 2.2	4.45 ± 2.1	3.92 ± 1.8	5.14 ± 2.7	5.4 ± 2.8	5.0 ± 2.7	4.23	0.015^c^
IBIL (μmol/L)	8.18 ± 4.4	8.86 ± 5.0	7.14 ± 3.9	9.0 ± 4.4	10.0 ± 7.7	8.80 ± 4.9	5.84	0.003^c^
TG (mmol/L)	1.08 ± 0.67	1.36 ± 1.0	1.58 ± 1.0	1.06 ± 0.6	1.23 ± 0.60	1.34 ± 0.90	22.1	<0.001^b^
CHOL (mmol/L)	4.08 ± 0.82	4.06 ± 0.99	4.15 ± 0.80	4.06 ± 0.8	4.22 ± 0.80	4.14 ± 0.90	0.29	0.75^c^
HDL-CH	1.24 ± 0.29	1.23 ± 0.36	1.14 ± 0.25	1.34 ± 0.5	1.24 ± 0.28	1.20 ± 0.27	5.96	0.003^c^
LDL-CH	2.45 ± 0.74	2.50 ± 0.9	4.16 ± 19	2.36 ± 0.6	2.56 ± 0.74	2.46 ± 0.87	0.99	0.372^c^
Chlorpromazine-equivalent dose (mg/day)	388.9 ± 198.8	408.0 ± 186.3	373.8 ± 153.2	257.1 ± 130.5	307.6 ± 189.2	312.0 ± 153.3	1.47	0.479^b^
AAPD regimens								
Olanzapine	29 (46.8%)	83 (44.6%)	36 (22.9%)	22 (38.6%)	34 (33.0%)	14 (18.4%)		
Risperidone	5 (8.06%)	15 (8.06%)	12 (7.64%)	8 (14.0%)	7 (6.80%)	12 (15.8%)		
Aripiprazole	1 (1.61%)	2 (1.08%)	3 (1.91%)	3 (5.26%)	2 (1.94%)	6 (7.89%)		
Clozapine	0	5 (2.69%)	15 (9.55%)	1 (1.75%)	3 (2.91%)	5 (6.58%)		
others	9 (14.5%)	22 (11.8%)	30 (19.0%)	11 (19.3%)	26 (25.0%)	16 (20.8%)		
Combination treatment	19 (30.6%)	54 (29.0%)	61 (38.9%)	12 (21.1%)	32 (31.1%)	24 (31.6%)		

The value was presented as *N*(%) or mean ± standard deviation.

*BMI* body mass index, *AAPD* atypical antipsychotic drug, *TBIL* total bilirubin, *DBIL* direct bilirubin, *IBIL* indirect bilirubin, *TG* triglyceride, *CHOL* cholesterol, *HDL-CH* high density lipoprotein cholesterol, *LDL-CH* Low density lipoprotein cholesterol;

*The *p* value less than 0.05 was considered significant.

^a^Analyzed by chi-squared test;

^b^by Kruskal–Wallis test

^c^by one-way ANOVA test.

### The relationship between bilirubin and lipids and their responses to AAPD

**Fig. 3:**
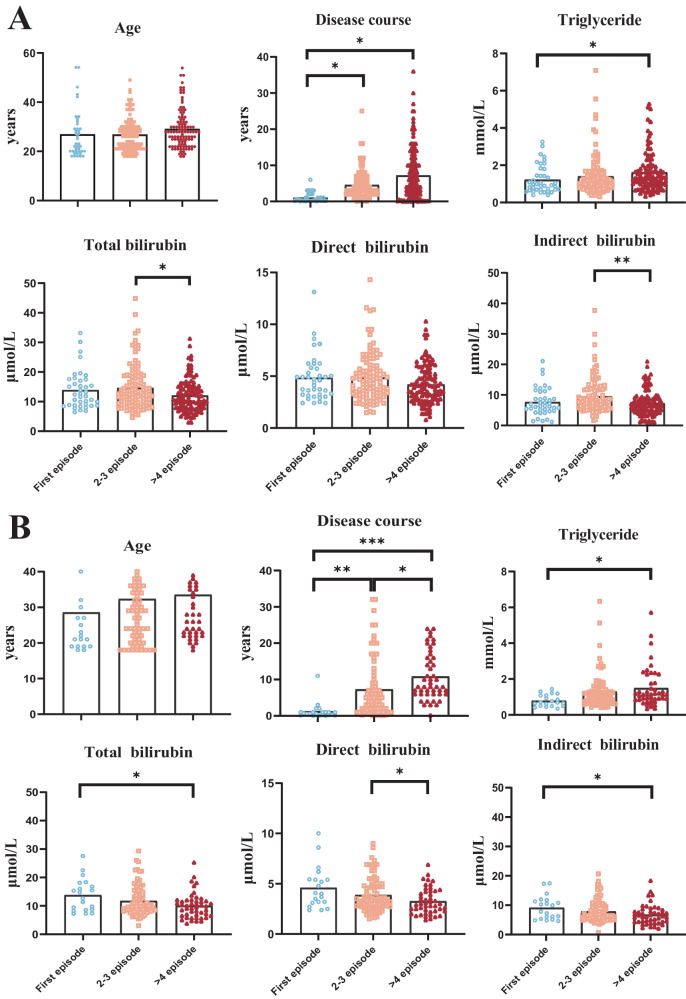
Comparison of demographic and biochemical parameters among subgroups in patients with schizophrenia from center 1. **A** Differences of age, disease course, TG, TBIL, DBIL, and IBIL from several episode subgroups in male patients with schizophrenia; **B** Differences of age, disease course, TG, TBIL, DBIL, and IBIL from several episode subgroups in female patients with schizophrenia. *N* (male) = 254, *N* (female) = 152. *p < 0.05, **p < 0.01.

Supplementary Fig. S1 displayed no significant differences in levels of bilirubin or lipid-related indexes among different patterns of AAPDs including monotherapy along with combination treatment from two centers. As shown in Fig. 3A (male cohort), the levels of TBIL and IBIL in the 2–3 subgroup were the highest, followed by the first-episode subgroup, and there was a significant difference between the 2–3 subgroup and the ≥4 subgroup (TBIL: ANOVA: F_(2,249)_ = 4.266, *p* = 0.015; 2–3 episodes vs. >4 episodes: *p* = 0.012; IBIL: ANOVA: F_(2,249)_ = 6.326, *p* = 0.002; 2–3 episodes vs. >4 episodes: *p* = 0.002). The TG of the first-episode subgroup, 2–3 subgroups, and ≥4 subgroups showed an increasing trend, and there was a significant difference in the TG between the first-episode subgroup and ≥4 subgroups (TBIL: ANOVA: F_(2,238)_ = 3.266, *p* = 0.04; First episode vs. >4 episodes: *p* = 0.041). Besides, Fig. 3B (female cohort) depicted that the levels of TBIL (ANOVA: F_(2,144)_ = 4.106, *p* = 0.018; First episode vs. >4 episodes: *p* = 0.018), DBIL (ANOVA: F_(2,144)_ = 5.163, *p* = 0.007; 2–3 episodes vs. >4 episodes: *p* = 0.006) and IBIL (ANOVA: F_(2,144)_ = 3.242, *p* = 0.042; First episode vs. >4 episodes: *p* = 0.038) decreased significantly as the number of episodes increased. In contrast, TG (ANOVA: F_(2,138)_ = 3.666, *p* = 0.028; First episode vs. >4 episodes: *p* = 0.021) showed a significantly increasing trend among the three subgroups.

### The impact of genotyping on bilirubin and lipids

In the study population, the whole-exome sequencing of PGRMC1 and HOMX1 indicated that their exon mutation frequencies of them were lower than 10%, and the mutation of UGT1A1*28 (rs34983651) was about 5%, which cannot be performed with valid statistical analysis due to the very small frequency of the mutation. On the contrary, the mutation frequency of rs2306283 polymorphism in SLCO1B1 was 96% in males and 94% in females, and that of the rs7566605 variant in INSIG2 was 85%. Therefore, these genes above were not included in the further analysis either. Among the gene polymorphisms of bilirubin metabolism in Supplementary Table S1, the mutation frequency of UGT1A1*6 and SLCO1B1*15 was high in males and females, moreover, the proportion of mutation in UGT1A1*6 (male 24.8%, female 29.9%) was more than that of SLCO1B1*15 (male 17.4%, female 14.4%). The mutation frequencies of INSIG1 and SREBF1 were both lower than 10% in male and female schizophrenia patients.

**Fig. 4:**
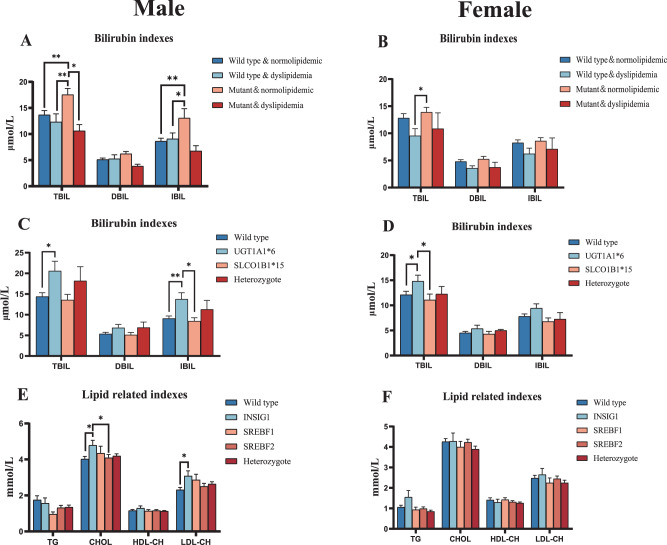
Effects of genotype related to bilirubin metabolism and lipid metabolism on biomedical parameters. **A** Genotypes and phenotypes of bilirubin with lipid metabolism in males; **B** Genotypes and phenotypes of bilirubin with lipid metabolism in females; Wild-type or mutant accord with whether mutations occur in gene polymorphisms related to bilirubin metabolism. Normolipidemic means patients without dyslipidemia; **C** Bilirubin level of each genotype related to bilirubin metabolism in male schizophrenics; **D** Bilirubin level of each genotype related to bilirubin metabolism in female schizophrenics; Wild-type means no mutation in genes related to bilirubin metabolism. UGT1A1*6 means the rs4148323 polymorphism (UGT1A1). SLCO1B1*15 means the rs4149056 and rs2306283 polymorphism (SLCO1B1); **E** Lipid level of each genotype related to lipid metabolism in male schizophrenics; **F** Lipid level of each genotype related to lipid metabolism in female schizophrenics. Wild-type means no mutation in genes related to lipid metabolism. INSIG1 means the rs9769826 polymorphism (INSIG). SREBF1 means the rs2297508 polymorphism (SREBF). SREBF2 means the rs1052717 polymorphism (SREBF); *N*(male) = 121, N(female) = 117. *p < 0.05; **p < 0.01.

Figure 4A shows the male patients with mutations in bilirubin-related polymorphism and normal lipid parameters were significantly higher than other subgroups in TBIL (ANOVA: F_(3,96)_ = 4.725, *p* = 0.004) and IBIL (ANOVA: F_(3,98)_ = 3.562, *p* = 0.017). In the genotype of bilirubin metabolism in male patients with schizophrenia (Fig. 4C), the TBIL level in patients with UGT1A1*6 (ANOVA: F_(3,109)_ = 3.675, *p* = 0.014; UGT1A1*6 vs. wild type: *p* = 0.016) was significantly higher than that in wild type patients. Besides, the IBIL levels of UGT1A1*6 type (ANOVA: F_(3,108)_ = 4.625, *p* = 0.004; UGT1A1*6 vs. wild type: *p* = 0.004; UGT1A1*6 vs. SLCO1B1*15: *p* = 0.032) were also significantly higher than those of wild type and SLCO1B1*15 type. Although the comparisons among the other subgroups were not significant, three bilirubin levels of UGT1A1*6, SLCO1B1*15, and heterozygous type were all higher than the wild type, among which the UGT1A1*6 was the highest. In the female schizophrenia patients (Fig. 4D), only the TBIL level of UGT1A1*6 (ANOVA: F_(3,108)_ = 4.625, *p* = 0.004; UGT1A1*6 vs. wild type: *p* = 0.004; UGT1A1*6 vs. SLCO1B1*15: *p* = 0.032) was significantly higher than the wild type and SLCO1B1*15. In addition, for the genotype of lipid metabolism in male schizophrenia patients (Fig. 4E), the level of CHOL in INSIG1 (rs9769826) genotype (ANOVA: F_(4,95)_ = 2.847, *p* = 0.028; INSIG1 vs. wild type: *p* = 0.023; INSIG1 vs. SREBF2: *p* = 0.046) was significantly higher than in wild type and the SREBF2 (rs1052717) genotype and the level of LDL-CH in INSIG1 (rs9769826) genotype (ANOVA: F_(4,96)_ = 3.003, *p* = 0.022; INSIG1 vs. wild type: *p* = 0.031) was significantly higher than wild type. However, in the female schizophrenia patients, the differences in lipid metabolism parameters among the five lipid metabolism-related genotypes did not approach significance.

### UGT1A1*6 as a protective factor for dyslipidemia induced by AAPD

**Fig. 5:**
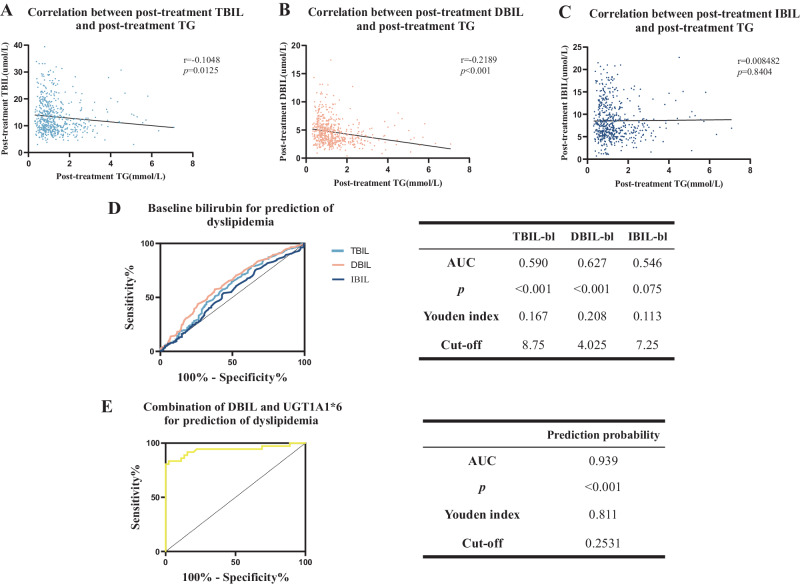
Correlation and prediction of the clinical data. The association between post-treatment bilirubin and post-treatment TG (**A**–**C**). Receiver-operating characteristic (ROC) curves of baseline TBIL, DBIL, and IBIL for prediction of antipsychotic-induced dyslipidemia (**D**). The combination of baseline level DBIL and whether the patient carries UGT1A1*6 mutation significantly improve the performance of prediction (**E**). The TBIL-bl, DBIL-bl, and IBIL-bl mean the bilirubin in the baseline.

As listed in Supplementary Table S2, in the mildly and moderately abnormal subgroup compared to severely abnormal subgroup, bilirubin metabolism-related gene mutations, especially the UGT1A1*6 mutation, were protective factors for lipid metabolism abnormalities. Additionally, we found that post-treatment TG was negatively associated with post-treatment TBIL (*r* = −0.1048, *p* = 0.0125) and post-treatment DBIL (*r* = −0.2189, *p* < 0.001), respectively (Fig. 5A, B). The association between post-treatment IBIL and post-treatment TG, however, was not significant (*r* = 0.008482, *p* = 0.8404) (Fig. 5C). What’s more, the ROC analysis indicated that the predictive performance of pre-treatment DBIL for antipsychotic-induced dyslipidemia was the best among the three bilirubin parameters, which still has an inadequate area under the curve (AUC) of 0.627 (Fig. 5D). When combining the information including the value of baseline DBIL and whether the patient possesses UGT1A1*6 mutation, the power of prediction can be greatly improved to an AUC of 0.939 (Fig. 5E).

## Discussion

The goal of antipsychotic drug treatment for schizophrenia is to achieve optimal symptomatic relief while minimizing side effects. To our knowledge, our study is the first to investigate the link between clinical manifestations, biochemical changes in bilirubin indexes and lipid-related indexes of different subgroups, and their associated genetic metabolic pathways in patients with schizophrenia. Moreover, our study translated biochemical levels and protective genotypes into predicting the risk of dyslipidemia. The present study confirmed that: (1) the association between bilirubin and TG was negative after medication, and (2) as the number of episodes went up, there were tendencies of bilirubin decrease and TG increase. Besides, (3) the mutation of UGT1A1*6 was significantly close to bilirubin levels and (4) UGT1A1*6 and the baseline bilirubin may be protective predictors for dyslipidemia.

The recombinant UDP glucuronosyltransferase 1 family, polypeptide A1 (UGT1A1), an important enzyme responsible for bilirubin biotransformation, has several SNPs such as UGT1A1*6 and UGT1A1*28^41^. Among them, the UGT1A1*6 (rs4148323) is a common mutant in Asia which leads to the decrease of UGT1A1 activity and abnormal bilirubin metabolism^42^, and our results were also based on the Chinese population, which means the findings are worthy of further exploration in other cohorts of different races. Several recent studies also suggested the indivisible association between serum bilirubin levels and UGT1A1*6 in the Asian population^43,44^. For example, a retrospective study in Japanese patients with chronic myeloid leukemia supported the proportion of patients with hyperbilirubinemia was significantly higher among patients with the UGT1A1*6/*6 and *6/*28 genotypes than among those with other genotypes^44^. However, the frequency of UGT1A1*6 mutations in other areas like European (G > A, G = 0.9986, A = 0.001446) or Africa (G > A, G = 0.9987, A = 0.0013) is low from the NCBI database of genetic variation (https://ncbi.nlm.nih.gov/snp/rs4148323). Interestingly, the serum bilirubin levels in these areas seem closer to the mutation of UGT1A1*28, another important haplotype from the UGT1A1 family. For example, a recent study in the northwest region of Russia cohort reported UGT1A1∗28 genotyping should be used as a prognostic risk factor for Gilbert’s Syndrome development^45^. The minimum allele frequencies of different UGT1A1 polymorphisms are discrepant in different races so that the status of bilirubin metabolism may be separate in every ethnic schizophrenia patient in response to the AAPD treatment. Thus, the AAPDs-induced metabolic side reactions have ethnic discrepancy and the prediction of genotyping should be discussed distinctly in different races.

As we know, the pathogenesis of schizophrenia often accompanied with increased oxidative stress^46,47^, and antipsychotic treatment may exert the antioxidative effect through increasing antioxidant substrates or lowering lipid peroxidation or NO production^48,49^. As a kind of strong antioxidant substrates, however, our results indicated that the bilirubin levels in schizophrenia patients still had a decreasing trend during a 4-week AAPD medication even if the psychotic symptoms were alleviated. In the disease progression period, it is indeed very hard to distinguish whether the levels of bilirubin changes are due to the schizophrenia itself or the antipsychotic treatment, and more informative biomarker should be obtained from those first-episode and drug-naïve patients with schizophrenia^50^. In support, Lu et al. found that the levels of bilirubin were reduced after antipsychotic treatment in first-episode and antipsychotic-naïve schizophrenia patients^51^. And a study by Meneca et al. also reported the significant reduction in baseline serum bilirubin from a cohort of first-episode schizophrenia patients in contrast to healthy controls^52^. In present study, we observed the same decreasing effects of AAPD on bilirubin levels both in the first-episode antipsychotic-naïve and relapsed antipsychotic-free schizophrenia subgroups, which suggests that abnormal lipid metabolism may already exist in antipsychotic-naive first-episode patients with schizophrenia and will be aggravated by AAPD treatment^53^. In addition, our results showed the significant decrease in bilirubin didn’t occur until over 4 episodes in male patients which was later than that in female patients. The sex difference in bilirubin indicated that female patients may be tougher to confront lipid peroxidation or in other words more sensitive to overweight or Mets. The numbers of studies were consistent with our result above^54,55^, for example, a previous study of 973 Chinese schizophrenia patients reported that females were almost twice as likely to be obese compared with males after antipsychotic drug therapy^56^. Interestingly, a recent study focused on first-episode drug-naïve schizophrenia patients supposed the opposite viewpoint that male patients had a greater prevalence of high BMI and hypertriglyceridemia than female patients^57^. The two reasons may account for that, firstly, the influence of AAPD is greater in the female metabolic system since AAPD-induced hyperprolactinemia will result in menstrual abnormalities^58^ and bilirubin decrease induced by AAPD is more susceptive in females. Secondly, the gender differences in metabolic risks among schizophrenia patients without antipsychotic exposure or general people are more based on sex hormones like estrogen which can reduce the formation and oxidation of lipid indexes^59^. However, the interaction mechanisms between the two patterns need further study in the future.

From the prospective cohort, our data demonstrated that the mutation of UGT1A1*6 was strongly related to the increase in the level of bilirubin as compared with the mutation of SLCO1B1*15. However, this may not be the case for patients other than schizophrenia. Usually, the high level of bilirubin is due to the combinational effects of several single nucleotide polymorphisms in the metabolic pathway of bilirubin. A recent genotype study showed that nearly 82% of cases possess four or more gene mutations like UGT1A1, SLCO1B1, HMOX1, and BLVRA in patients with hyperbilirubinemia^60^. In the lipid metabolism, studies reported that the four protein complexes including SREBP, SCAP, INSIG, and PGRMC1 in the endoplasmic reticulum may be the important regulators of dyslipidemia caused by AAPD^12,61^. Among them, the protein INSIG forms complexes with SREBPs and SCAP to regulate the biosynthesis of CHOL and fatty acids^62^. The results in the present study indicated that the mutation of INSIG1 rs9769826 may aggravate the dyslipidemia, especially for increased CHOL in male patients with schizophrenia than female. In animal studies, the overexpression of INSIG1 gene reduced high levels of lipid parameters in both liver and plasma of Zucker rats^63^, while single knockout of either INSIG1 or INSIG2 in mice led to an increment of the total content of cholesterol in mouse livers^64^. From this perspective, the polymorphism of INSIG1 in our study may imply that patients possess the silent gene will likely be accompanied with elevated CHOL levels. In support, a clinical study in Xinjiang, China reported that rs2721 and rs9719268 of the INSIG1 gene were associated with an increased risk of obesity^65^. For sex differences in INSIG1 polymorphism induced high CHOL, the sex-biased gene expression may explain that^66^. A study by De Marinis E, et al found that higher INSIG expression (INSIG1 and INSIG2) and lower SREBP expression (nSREBP2 and SREBP2) were observed in female rats than male rats^67^. Since higher INSIG expression is theoretically associated with reduced lipid levels, it could be explainable that lower expression combined with the recessive mutation of INSIG1 may have the elevated CHOL levels specifically in male patients.

In the multinomial logistic regression analysis, we found that the gene mutation about bilirubin metabolism especially UGT1A1*6 plays a protective role in the lipid metabolism of schizophrenia patients after treatment. A study found that the TG and CHOL of the Gilbert syndrome population were significantly lower than those of the normal population^68^, and it was found that the homozygous recessive Gunn rats (UGT1A1 deficiency resulted in higher bilirubin levels in the body, and the homozygous recessive rats had the highest bilirubin levels), and the body weight is also lighter than that of the littermates^69^, which indicates that the elevated bilirubin level caused by the mutation of the bilirubin metabolism-related gene can prevent the lipid metabolism disorder to a certain extent. Among male patients with schizophrenia, the population with bilirubin gene mutations but abnormal lipid metabolism is only about 50% of the wild type. While there is no phenomenon among female schizophrenia patients. In other words, the correlation between changes in lipid metabolism and the level of bilirubin may be closer in male schizophrenia patients. The result of genotype and phenotype in Fig. 4A showed that baseline bilirubin levels were the highest in male patients with bilirubin gene mutations but normal lipid metabolism. However, there were no significant differences in the other three subgroups. Similar results could also be obtained from female patients which indicated that the schizophrenia patients with variants in bilirubin metabolism but without the increased bilirubin levels were unable to resist the metabolic side effects after AAPD. Therefore, the variants in bilirubin metabolism and certain levels of bilirubin may be crucial protective factors for dyslipidemia induced by AAPD. Based on that, the findings pushed us to explore the predictive value of dyslipidemia from the above two indicators.

We conducted the ROC analysis to explore the good/poor prediction by three bilirubin on dyslipidemia. The result showed DBIL had the best prediction among the three bilirubin (Fig. 5D), which is not consistent with Fig. 4A (Only the baseline TBIL and IBIL of mutant & normolipidemic individuals were higher than other groups significantly). There are two reasons accounting for that: (1) The unconjugated bilirubin (IBIL) can transform into conjugated bilirubin (DBIL) in the endoplasmic reticulum through the enzyme UGT1A1, however mutations in UGT1A1 can lead to decreased expression or inactivation of the enzyme so that the baseline IBIL in the mutant individuals are more prone to accumulate than others and the baseline TBIL also stay high^70^. (2) DBIL has a stronger antioxidative effect than TBIL and IBIL in dyslipidemia. It was reported that DBIL is more loosely bound to albumin and, as a result, more easily separated and in the active form^71^. A study in the Chinese cohort has confirmed that DBIL, not IBIL is negatively correlated with metabolic side effects^72^. In addition, IBIL demonstrated a stronger association with albumin compared to DBIL. This tighter conjunction with albumin suggests a potential competition with free drugs, leading to an increase in the concentration of AAPD. Conversely, elevated levels of DBIL in the bloodstream resulted in reduced IBIL levels and the following IBIL-albumin binding, allowing for greater interaction between drugs and albumin. This phenomenon may contribute to a decrease in the free fraction of AAPD in the serum, potentially serving as a protective mechanism against AAPD-induced dyslipidemia facilitated by elevated physiological levels of DBIL. When combining the DBIL and UGT1A1*6 as a new parameter to predict dyslipidemia, the power of prediction will be significantly improved (AUC from 0.627 to 0.939, Fig. 5E). The result of ROC analysis supported the connection between DBIL and UGT1A1 from another angle, and their regulatory effects on dyslipidemia. So based on our findings, we can expect that when patients with the mutation of UGT1A1*6 and has a DBIL level greater than 4 μmol/L, they may have a certain natural resistance to dyslipidemia induced by antipsychotic drugs. Except for dyslipidemia, UGT1A1 may also be related to other side reactions. For example, a random crossover clinical study reported that the UGT1A1 rs887829 is close to the changes in blood glucose after olanzapine medication^73^. In addition, the polymorphic variant rs887829, rs10929302, and rs111741722 of UGT1A1 were significantly associated with hyperprolactinemia in a study from Thailand^74^. Consequently, the significance and mechanism of UGT1A1 in the metabolic status of antipsychotic drugs remain to be further explored.

There were several limitations in our study to be noted. Firstly, we only studied the major genes on the metabolic pathway of bilirubin such as UGT1A1 and SLCO1B1, which is due to the low frequency or correlation of other mutations as reported previously^75–77^. Thus, it is urgent to expand the screening of more bilirubin-related gene polymorphisms in the future. Moreover, as the target population for genotyping, the sample size of center 2 is still small. Whereas the patient cases from center 2 in our study were mainly used to confirm the results of center 1 and for a prospective study. Therefore, a larger sample size should be included in further study. Thirdly, it is important to note that the evidence in the specific context of our study might not be as robust as in other areas of pharmacogenetics. Compared to previous pharmacogenomic/pharmacogenetic studies which lay more emphasis on the influence of the genetic variations on both the pharmacokinetics and pharmacodynamics of antipsychotic drugs, the present study was mainly focusing on the target pathway related to bilirubin and lipid metabolism that is impacted by antipsychotic drugs, pharmacogenetic techniques seemed to be more appropriate. Finally, chronic patients with multiple episodes were enrolled in the study, so it may be hard to distinguish whether the changes in bilirubin were fully due to AAPDs or partially owing to the progression of schizophrenia pathophysiology. However, we also observed the same decreasing effects of AAPD on bilirubin levels in the first-episode antipsychotic-naïve schizophrenia subgroup, which suggests that it is more related to the action of antipsychotics. In future studies, it may be more informative to monitor the level changes of bilirubin during the unmedicated period of schizophrenia patients in the first episode before the initiation of antipsychotic treatment.

In conclusion, the level of serum bilirubin and parameters in lipid metabolism especially TG and CHOL showed a negative correlation in schizophrenia patients after the medication of AAPD. Elevation of serum direct bilirubin (DBIL) within the physiological range, coupled with the presence of the UGT1A1*6 mutation in Chinese patients with schizophrenia, may serve as potential protective factors against AAPD-induced dyslipidemia. In the future, it’s essential to take on further research regarding the underlying mechanism of the protective effect of bilirubin on dyslipidemia. Besides, the mutation of UGT1A1*6 in Chinese patients could be equal to the mutation of UGT1A1*28 in the European and American populations, which suggests that metabolic side effects of AAPD might differ amongst racial and ethnic groups.

### Supplementary information

Supplementary material

## Data Availability

The data that support the findings of this study are available from the corresponding author upon reasonable request.
