# Supplementary material for: Combination of UGT1A1 polymorphism and baseline plasma bilirubin levels in predicting the risk of antipsychotic-induced dyslipidemia in schizophrenia patients
Source: Schizophrenia (Heidelb). 2024 May 17;10(1):52. doi: 10.1038/s41537-024-00473-1 (PMC11101411; doi:10.1038/s41537-024-00473-1)
Supplement: Supplementary file 1 — Supplementary material [file 41537_2024_473_MOESM1_ESM.docx]

***Supplementary material***

Combination of UGT1A1 polymorphism and baseline plasma bilirubin levels in predicting the risk of antipsychotic-induced dyslipidemia in schizophrenia patients

**Chenquan Lin, Shuangyang Zhang, Ping Yang, Bikui Zhang, Wenbin Guo, Renrong Wu, Yong Liu, Jianjian Wang, Haishan Wu, Hualin Cai^*^**

*** Correspondence: Prof. HuaLin Cai (hualincai@csu.edu.cn)**

**Supplementary Table 1.** List of gene mutations

**Supplementary Figure 1.** The differences of effects from several patterns of AAPD

**Supplementary Table 2.** Logistic regression analysis between potential factors and dyslipidemia

**Supplementary Table 3.** Baseline biochemical characteristics of schizophrenia patients from two center

**Supplementary Table 1. List of gene mutations**

| **Single Nucleotide Polymorphisms** | | | | |  | **Genotyping** | | | |
| --- | --- | --- | --- | --- | --- | --- | --- | --- | --- |
| **Name** | **SNP** | **High>Low** | **Male** | **Female** |  | | **Name** | **Male** | **Female** |
| PGRMC1 | EXON2 |  | 1(0.8%) | 2(1.71%) |  | | **Genes related to bilirubin metabolism** |  |  |
| HOMX1 | EXON1 |  | 5(4.1%) | 12(10.3%) |  | | Wild-type^b^ | 78(64.5%) | 69(59.0%) |
|  | EXON2,3,4^a^ |  | 4(3.3%) | 0 |  | | UGT1A1*6 | 22(18.2%) | 31(26.5%) |
| UGT1A1 | rs34983651 (UGT1A1*28) | (TA)_5/6_>(TA)_7/8_ | 8(6.6%) | 5(4.3%) |  | | SLCO1B1*15 | 13(10.8%) | 14(12.0%) |
|  | rs4148323 (UGT1A1*6) | G>A | 30(24.8%) | 35(29.9%) |  | | Heterozygous type^c^ | 8(6.61%) | 3(2.56%) |
| SLCO1B1 | rs2306283(SLCO1B1*1B) | A>G | 117(97%) | 110(94%) |  | | **Genes related to lipid metabolism** |  |  |
|  | rs4149056+ rs2306283 (SLCO1B1*15) | T>C | 21(17.4%) | 17(14.5%) |  | | Wild-type^d^ | 32(26.4%) | 37(31.6%) |
| INSIG1 | rs9769826 | A>G | 30(24.8%) | 25(21.4%) |  | | INSIG1 | 7(5.79%) | 9(7.69%) |
| INSIG2 | rs7566605 | C>G | 102(84%) | 100(85.5%) |  | | SREBF1 | 9(7.44%) | 10(8.55%) |
| SREBF1 | rs2297508 | G>C | 38(31.4%) | 25(21.4%) |  | | SREBF2 | 33(27.3%) | 36(30.8%) |
| SREBF2 | rs1052717 | A>G | 68(56.2%) | 59(50.4%) |  | | Heterozygous type^e^ | 40(33.0%) | 25(21.4%) |

The values were presented as N (%). a. The number of mutations in EXON2, EXON3, and EXON4 in HMOX1 in male schizophrenia is 1, 2 and 1; b. Wild-type means no mutation in genes related to bilirubin metabolism; c. Heterozygous type means both UGT1A1*6 and SLCO1B1*15 have mutated; d. Wild-type means no mutation in genes related to lipid metabolism; e. Heterozygous type refers to mutations in two or more genes of INSIG1, INSIG2, SREBF1 and SREBF2.


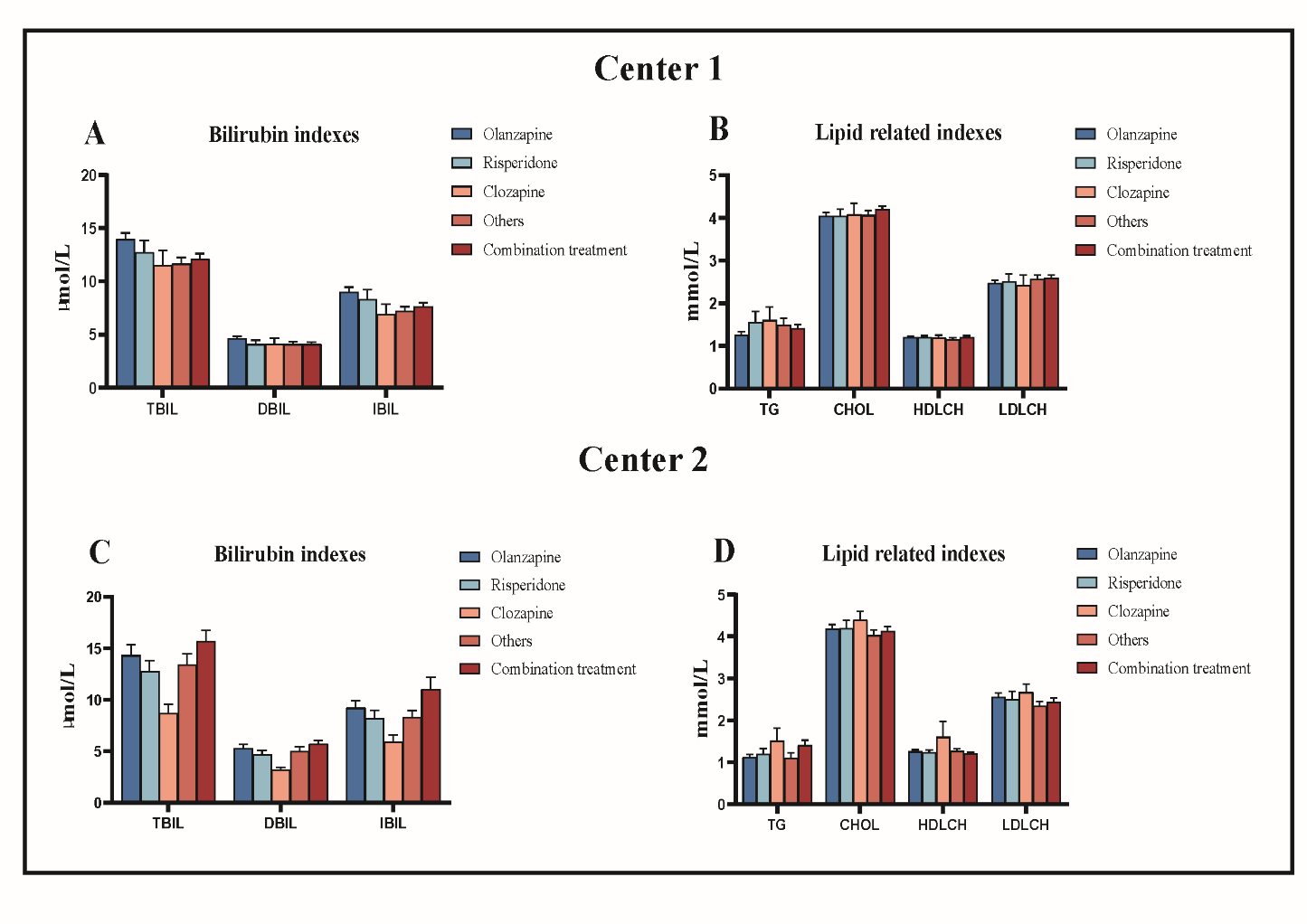


**Supplementary Figure 1. The differences of effects from several patterns of AAPD on (A, bilirubin indexes from center 1; B, lipid related indexes from center 1; C, bilirubin indexes from center 2; D, lipid related indexes from center 2).** AAPD, atypical antipsychotic drug; TBIL, total bilirubin; DBIL, direct bilirubin; IBIL, indirect bilirubin; TG, triglyceride; CHOL, cholesterol; HDL-CH, high density lipoprotein cholesterol; LDL-CH, Low density lipoprotein cholesterol. N (Center 1) =406, N (Center 2) =238. *p < 0.05, **p < 0.01.

**Supplementary Table 2.** **Logistic regression analysis between potential factors and dyslipidemia.**

|  | Factors | ß | *p* | OR (95%CI) |
| --- | --- | --- | --- | --- |
| Normal^a^ | TBIL | 7.625 | 0.211 | 2048.614(0.013- 3.16×10^8^) |
|  | DBIL | -8.654 | 0.159 | 0.000(0.000-29.835) |
|  | IBIL | -6.801 | 0.260 | 0.001(0.000-151.692) |
|  | Gender (male *vs* female) | 80.647 | 0.946 | 1.1×10^35^(0.000- d) |
|  | UGT1A1*6 (no *vs* yes) | -12.589 | 0.360 | 0.000(0.000-1.76×10^6^) |
|  |  |  |  |  |
| Mild | TBIL | -0.073 | 0.970 | 0.930(0.022-39.806) |
|  | DBIL | -0.545 | 0.771 | 0.580(0.015-22.923) |
|  | IBIL | 0.506 | 0.796 | 1.659(0.036-76.814) |
|  | Gender (male *vs* female) | 4.598 | <0.001 | 99.267(8.592-1146.919) |
|  | UGT1A1*6 (no *vs* yes) | -12.868 | <0.001 | 2.58×10^-6^(1.99×10^-8^-0.000) |
|  |  |  |  |  |
| Moderately | TBIL | -3.983 | 0.311 | 0.019(0.000-41.198) |
|  | DBIL | 3.755 | 0.339 | 42.744(0.019-9.42×10^4^) |
|  | IBIL | 4.214 | 0.285 | 67.631(0.030-1.52×10^5^) |
|  | Gender (male *vs* female) | 0.949 | 0.316 | 2.584(0.404-16.521) |
|  | UGT1A1*6 (no *vs* yes) | -14.556 | <0.001 | 4.77×10^-7^(1.33×10^-8^-1.71×10^-5^) |

**a.** no changes in lipid metabolism parameters; **b.** one lipid metabolism parameter meets the dyslipidemia criteria; **c.** two lipid metabolism parameters meet the dyslipidemia criteria; The reference category of **a.**, **b.** and **c.** is severe abnormal which carries three or more parameters meet the dyslipidemia criteria. **d.** Floating point overflow occurred while computing this statistic. Its value is therefore set to system missing.

**Supplementary Table 3. Baseline biochemical characteristics of schizophrenia patients from two center**

| **Indexes** | **Overall** | **Center 1** | **Center 2** |
| --- | --- | --- | --- |
| **ALT (U/L)** | 16 (11, 27) | 15 (10, 27) | 18 (12, 27) |
| **AST (U/L)** | 19 (16, 24) | 19 (15, 24) | 20 (16, 25) |
| **AST/ALT** | 1.17 (0.83, 1.59) | 1.20 (0.82, 1.63) | 1.15 (0.85, 1.52) |
| **TP (g/L)** | 68.9 (65.0, 72.7) | 67.6 (64.0, 71.0) | 71.5 (68.2, 76.6) |
| **ALB (g/L)** | 42.3 (40.0, 44.9) | 41.7 (39.5, 43.9) | 43.9 (40.9, 46.5) |
| **GLB (g/L)** | 26.7 (24.2, 28.8) | 25.8 (23.4, 27.6) | 27.8 (25.9, 30.6) |
| **A/G** | 1.60 (1.46, 1.78) | 1.63 (1.49, 1.81) | 1.56 (1.43, 1.70) |
| **TBIL (μmol/L)** | 12 (9, 16) | 11 (8, 16) | 12 (9, 18) |
| **DBIL (μmol/L)** | 4.10 (3.00, 5.50) | 3.80 (2.80, 5.30) | 4.80 (3.44, 6.00) |
| **IBIL (μmol/L)** | 7.2 (5.2, 10.5) | 6.9 (5.0, 9.8) | 7.8 (5.8, 11.5) |
| **TBA** | 3.8 (2.4, 6.6) | 4.0 (2.5, 6.8) | 3.6 (2.1, 6.4) |
| **TG (mmol/L)** | 1.08 (0.77, 1.56) | 1.11 (0.77, 1.62) | 1.00 (0.77, 1.42) |
| **CHOL (mmol/L)** | 3.99 (3.55, 4.59) | 3.97 (3.50, 4.56) | 4.00 (3.60, 4.61) |
| **HDL-CH (mmol/L)** | 1.17 (1.01, 1.35) | 1.15 (1.01, 1.34) | 1.23 (1.04, 1.38) |
| **LDL-CH (mmol/L)** | 2.35 (1.97, 2.92) | 2.38 (1.98, 2.94) | 2.28 (1.97, 2.87) |

The value was presented as N(%) or Median (interquartile range); Abbreviations: ALT, alanine aminotransferase; AST, aspartate aminotransferase; TP, total protein; ALB, albumin; GLB, globulin; TBA, total bile acid; TG, triglyceride; CHOL, cholesterol; HDL-CH, high density lipoprotein cholesterol; LDL-CH, low density lipoprotein cholesterol.
